# Processing of translational, radial and rotational optic flow in older adults

**DOI:** 10.1038/s41598-023-42479-2

**Published:** 2023-09-15

**Authors:** Jade Guénot, Yves Trotter, Angélique Delaval, Robin Baurès, Vincent Soler, Benoit R. Cottereau

**Affiliations:** 1 Brain and Cognition Research Center, Université Toulouse III - Paul Sabatier, Toulouse, France; 2 Centre National de la Recherche Scientifique, CNRS UMR5549, Toulouse, France; 3 Hôpital Purpan, Unité de Rétine - CHU Toulouse, Toulouse, France

**Keywords:** Cognitive ageing, Cognitive neuroscience, Human behaviour

## Abstract

Aging impacts human observer’s performance in a wide range of visual tasks and notably in motion discrimination. Despite numerous studies, we still poorly understand how optic flow processing is impacted in healthy older adults. Here, we estimated motion coherence thresholds in two groups of younger (age: 18–30, n = 42) and older (70–90, n = 42) adult participants for the three components of optic flow (translational, radial and rotational patterns). Stimuli were dynamic random-dot kinematograms (RDKs) projected on a large screen. Participants had to report their perceived direction of motion (leftward versus rightward for translational, inward versus outward for radial and clockwise versus anti-clockwise for rotational patterns). Stimuli had an average speed of 7°/s (additional recordings were performed at 14°/s) and were either presented full-field or in peripheral vision. Statistical analyses showed that thresholds in older adults were similar to those measured in younger participants for translational patterns, thresholds for radial patterns were significantly increased in our slowest condition and thresholds for rotational patterns were significantly decreased. Altogether, these findings support the idea that aging does not lead to a general decline in visual perception but rather has specific effects on the processing of each optic flow component.

## Introduction

The aging population is growing worldwide. According to the World Health Organization projections, the number of people over sixty will double by 2050, reaching 2.1 billions^1^. It therefore became critical for society to better understand the consequences of healthy aging on cognitive abilities and a growing number of studies characterized functional changes across lifespan over the last decades, with some heterogeneity in the reported results^2,3^. In the domain of sensory perception, many research teams have been interested in age effects on the processing of motion (see Billino & Pilz^4^ for a review) and notably of optic flow, the projection of the visual scene on the retina during locomotion. Optic flow is essential for heading^5,6^, collision detection^7^ and path integration^8^. Moreover, it is particularly relevant for the older adults who mostly rely on visual cues during navigation^9–11^.

Optic flow can be decomposed into different patterns (translational and rotational patterns, both due to eye movement and/or head rotation, and radial due to observer forward/backward displacements) that are processed by distinct neural populations along the visual system hierarchy^12,13^. So far, the majority of studies explored how age affects the perception of translational motion, notably in discrimination tasks (e.g., the discrimination between leftwards versus rightward motion) based on random-dot kinematogramms (RDKs) with varying signal-to-noise ratios. Most of these studies reported that translation perception is impaired in older adults (i.e., people over the age of 60)^14–16^, especially after 70 years old^15,17,18^. Different visual parameters can however modulate this deficit (see Billino & Pilz^4^ for a review): the luminance contrast of the dots^19^, the size of the stimulus^20^ or even its speed, with more pronounced deficits for slower stimuli (i.e., lower than 2°/s^21^).

Much fewer studies investigated the consequences of age on radial and rotational optic flow patterns, with disparate results. Using RDKs, Billino et al.^14^ found that heading perception from radial patterns remained stable across lifespan. Atchley and Andersen^22^ did not observe any significant effect of age for radial pattern, nor any correlation between thresholds for translational and radial motions, in line with the idea that the neural populations processing the two types of patterns differ. Conversely, Warren et al.^23^ found a general decline in the ability to detect global optic flow, with a small but significant increase in heading detection thresholds in older adults. Using a virtual reality set-up, Lich and Bremmer^24^ reported that older participants made more errors in absolute heading judgments based on radial flow. If other studies found that heading perception was impaired in older adults^25–27^, the reported effects were often limited^28,29^. To our knowledge, age effects on the processing of rotational patterns was only explored in one single study based on a limited number of participants^19^ and which found that discrimination thresholds for this type of motion (and also for translational and radial motion) were significantly higher in older adults but mostly at low contrasts. However, stimuli in this study did not contain velocity gradients (dots moved at a constant speed) and thus reflected global motion rather than optic flow.

To date, the effects of aging on the processing of the three optic flow components are not fully understood. Here, we estimated motion coherence thresholds and reaction times for these three components in two large groups of younger and older adult participants (84 subjects in total) using a curved screen that covered an important part of the visual field to reproduce optic flow patterns falling on the retina during navigation. Because it was reported that motion discrimination performances in older adults are specifically reduced in central vision^30^, a secondary objective was to characterize the same processing when only the peripheral vision was stimulated. Thus, thresholds and reaction times were also estimated among the same participants using a simulated scotoma that covered the central 20° of their visual field (see the "Methods" section). We reproduced all of these measurements at a different speed as this variable was reported to modulate performances in older participants^4,21^. Finally, we also looked for gender differences in our data as previous studies found that age effects on motion processing are more pronounced in women (see Hutchinson et al.^31^ and Billino & Pilz^4^ for reviews). The experimental protocol was similar to the one of our previous study on optic flow processing in patients with macular degeneration^32^. We notably used an adaptive Bayesian psychophysical procedure to estimate robust motion coherence thresholds in our participants.

## Results

**Figure 1:**
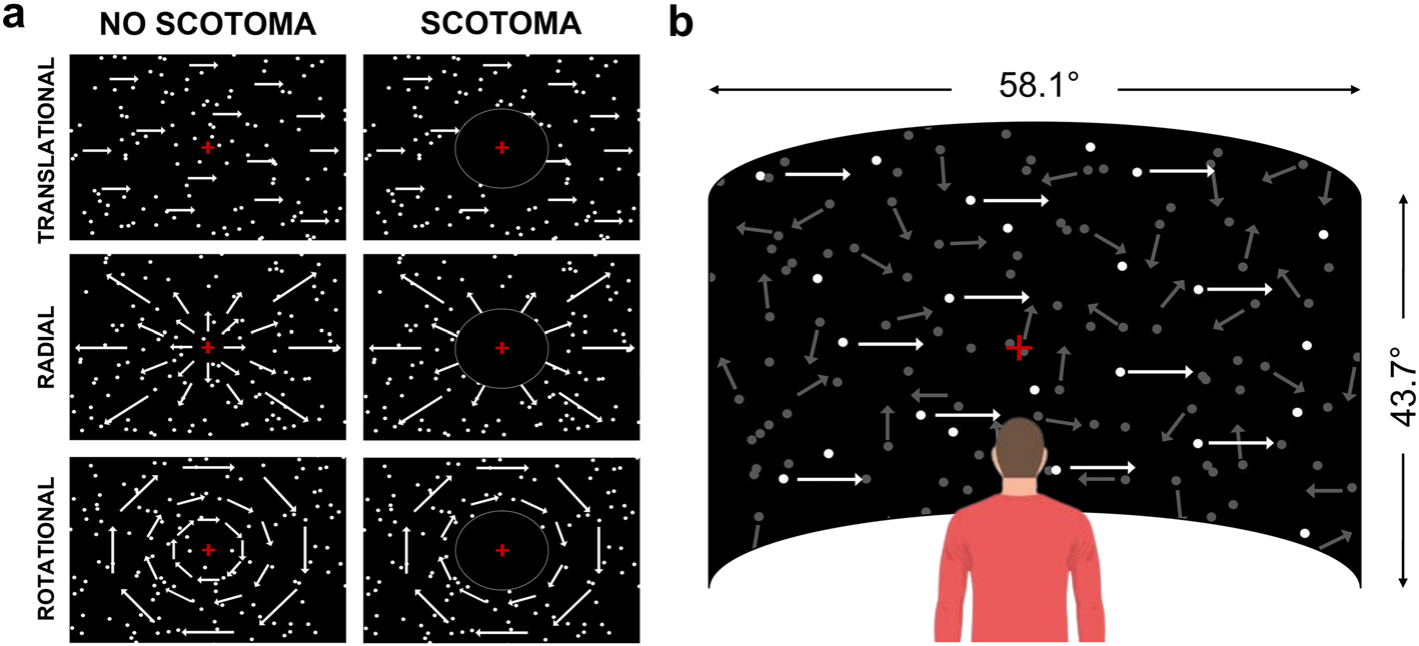
Stimuli and viewing conditions. (**a**) The three components of optic flow (translational, radial and rotational) were defined from RDKs. We used two different viewing conditions: full-field (left column) and peripheral vision (with an artificial scotoma that covered the 20° of the central visual field°, right column). Note that the scotoma is circled in white for illustration only. (**b**) RDKs were projected on a convex screen at a viewing distance of 180 cm. Younger (n = 42) and older (n = 42) adult participants had to fixate on the central red cross and to report the perceived motion direction of the stimuli. We manipulated motion coherence and estimated thresholds corresponding to 80% of correct detections. For illustration, a translational trial is shown at 20% of coherence. Signal dots are shown in white and noise dots in gray for better visibility on the figure (all the dots were white during the experiment).

The main objective of this study was to characterize how age impacts optic flow processing. In two groups of younger (age: 18–30, n = 42) and older (age: 70–90, n = 42) adult participants, we estimated coherence thresholds and reaction times for the three patterns of optic flow (translational, radial and rotational) during a motion direction discrimination task with an average dot speed of 7°/s (see Fig. 1).

A secondary objective was to characterize how the absence of central vision impacts the same processing among the same participants. Thus, thresholds and reaction times were also estimated while the central 20° of the screen was masked by a simulated scotoma.

We performed additional recordings with an average dot speed of 14°/s in a subgroup of 23 younger (16 women, mean age = 23.3 ± 2.61) and 23 older (13 women, mean age = 73.3 ± 4.53) adult participants (see the "Methods" section).

### Age effects on the processing of optic flow patterns in younger and older participants

#### Age effects on motion direction thresholds

**Figure 2:**
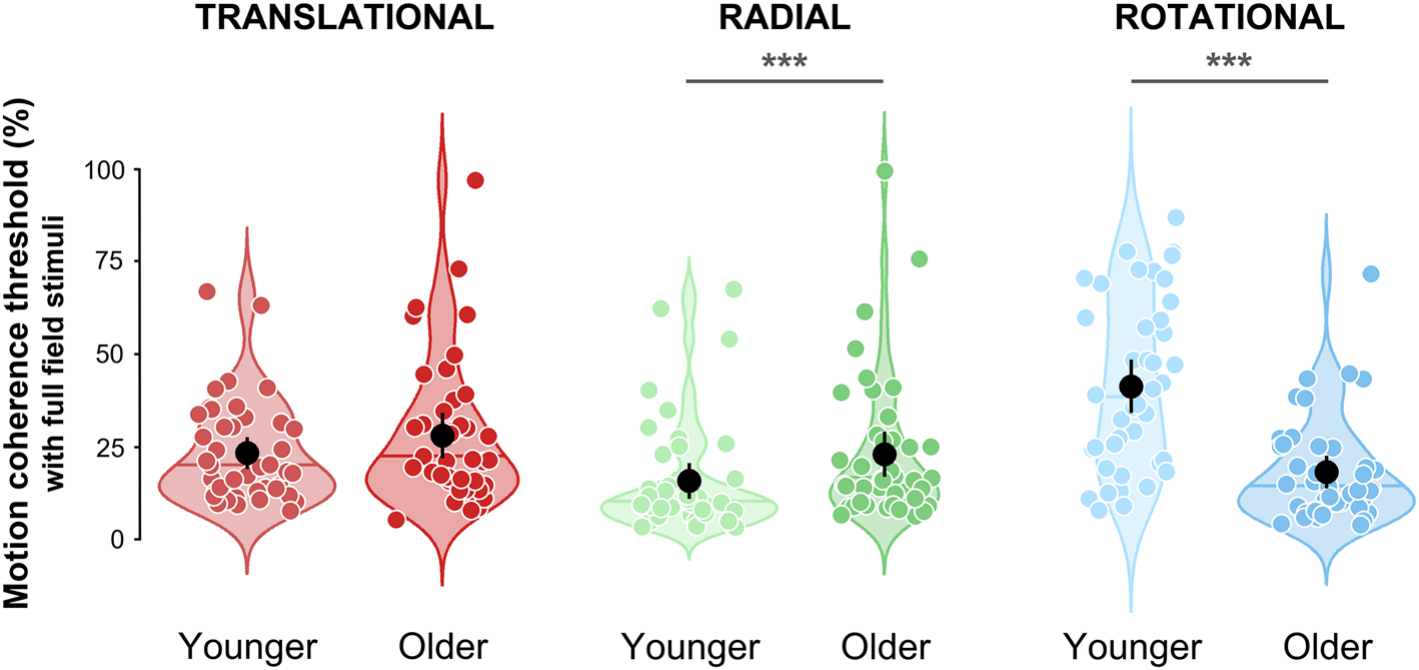
Distributions of the motion coherence thresholds estimated for full-field translational (red), radial (green), and rotational (blue) optic flow patterns, for the younger (age: 18–30, n = 42, light colors) and older (age: 70–90, n = 42, dark colors) adults groups. Black dots represent group-level means. Error bars represent the associated 95 percent confidence intervals. Horizontal colored lines represent group-level medians. Circles provide the individual data points of the distributions. Note that they were slightly offset horizontally to improve their visibility. Stars indicate significantly different distributions (***p < 0.001).

Figure 2 shows the distribution of motion coherence thresholds in the two groups for the three components of optic flow. The data are presented here for the full-field condition (i.e., without a central scotoma). Lower thresholds correspond to better discrimination performances. Distributions of the data collected for the peripheral vision condition (i.e., when stimuli were masked with a central scotoma) are provided in Supplementary Text S1 and Fig. S1.

We ran a two-way ANOVA with the group (young or older participants) and the gender (women or men) as between factors and the optic flow pattern (translational, rotational or radial) and the viewing condition (full-field or peripheral vision) as within factors. This ANOVA led to significant effects of the optic flow pattern (F(2, 160) = 24.58, p < 0.001, η_p_^2^ = 0.237). Post-hoc t-tests (with Welch’s adjustment for variance inequality) corrected for multiple comparisons (Bonferroni) demonstrated that thresholds were significantly lower for radial patterns (mean ± s.d. radial = 19.3 ± 15.6%) than for rotational (mean rotational = 29.9 ± 21.6%, p < 0.001, *d* = 0.584) and translational patterns (mean translational = 25.9 ± 16.3%, p < 0.001, *d* = 0.581), without difference between the latter patterns (p = 1.0, *d* = 0.075). The ANOVA showed no effect of the group alone (F(1, 80) = 0.19, p = 0.662, η_p_^2^ = 0.002) but the interaction between the groups and the optic flow pattern was also significant (F(2, 160) = 45.85, p < 0.001, η_p_^2^ = 0.364). Post-hoc t-tests corrected for multiple comparisons indicated that thresholds were lower in the younger group for radial patterns (mean radial = 15.7 ± 13.0% for younger and mean radial = 22.9 ± 17.2% for older participants, t(163.98) = 4.14, p < 0.001, *d* = 0.638) and higher in the younger group for rotational patterns (mean rotational = 40.8 ± 22.9% for younger and mean rotational = 19.1 ± 13.2% for older participants, t(16.73) = 7.70, p < 0.001, *d* = 1.188, see Fig. 2). Coherence thresholds were not significantly different between groups for the translational patterns (mean translational = 23.9 ± 14.2% for younger and mean translational = 27.8 ± 18.1% for older participants, t(165) = 1.44, p = 0.151, *d* = 0.223). Post-hoc pairwise t-tests also revealed that younger participants had higher motion coherence thresholds for rotational than for translational (p < 0.001, *d* = 0.807) and radial thresholds (p < 0.001, *d* = 1.496), and for translational than for radial thresholds (p < 0.001, *d* = 0.808). Older participants had higher thresholds for translational than radial patterns (p = 0.007, *d* = 0.369) or than rotational patterns (p < 0.001, *d* = 0.649), with no difference between the two latter patterns (p = 0.084, *d* = 0.274). Moreover, no gender effect was found (F(1, 80) = 2.21, p = 0.141, η_p_^2^ = 0.030) but the interaction between gender and optic flow pattern was significant (F(2,160) = 3.46, p = 0.034, η_p_^2^ = 0.031, see Supplementary Fig. S2). Post-hoc t-tests notably showed that women had higher thresholds than men for translational patterns only (mean women = 29.1 ± 15.8%, mean men = 22.4 ± 16.3%, t(81.92) = 3.27, p = 0.002, *d* = 0.696). Note that here, gender has also been taken into consideration as several studies found an effect of this variable^4,31^. Further details are provided in Supplementary Text S2.

As a secondary outcome, the ANOVA also led to a significant effect of the viewing condition, F(1, 80) = 7.85, p = 0.006, η_p_^2^ = 0.079 (mean full-field = 24.9 ± 19.6%, mean peripheral vision = 25.1 ± 17.4%), but no significant interaction between this factor and the pattern or the group was established (respectively F(2, 160) = 0.59, p = 0.558, η_p_^2^ = 0.007 and F(1, 80) = 0.91, p = 0.344, η_p_^2^ = 0.012). Supplementary Fig. S1 supplies detailed data.

Two older adult participants had motion discrimination thresholds near 100%, one for the translational and the other for the radial patterns (see Fig. 2). These two participants were nonetheless able to perform the task because they obtained valid thresholds for the two other patterns. To make sure that the data associated with these two subjects did not impact our results, we reproduced the statistical analyses described above using only the other participants and found that our conclusions remained unchanged.

#### Age effects on reaction times

**Figure 3:**
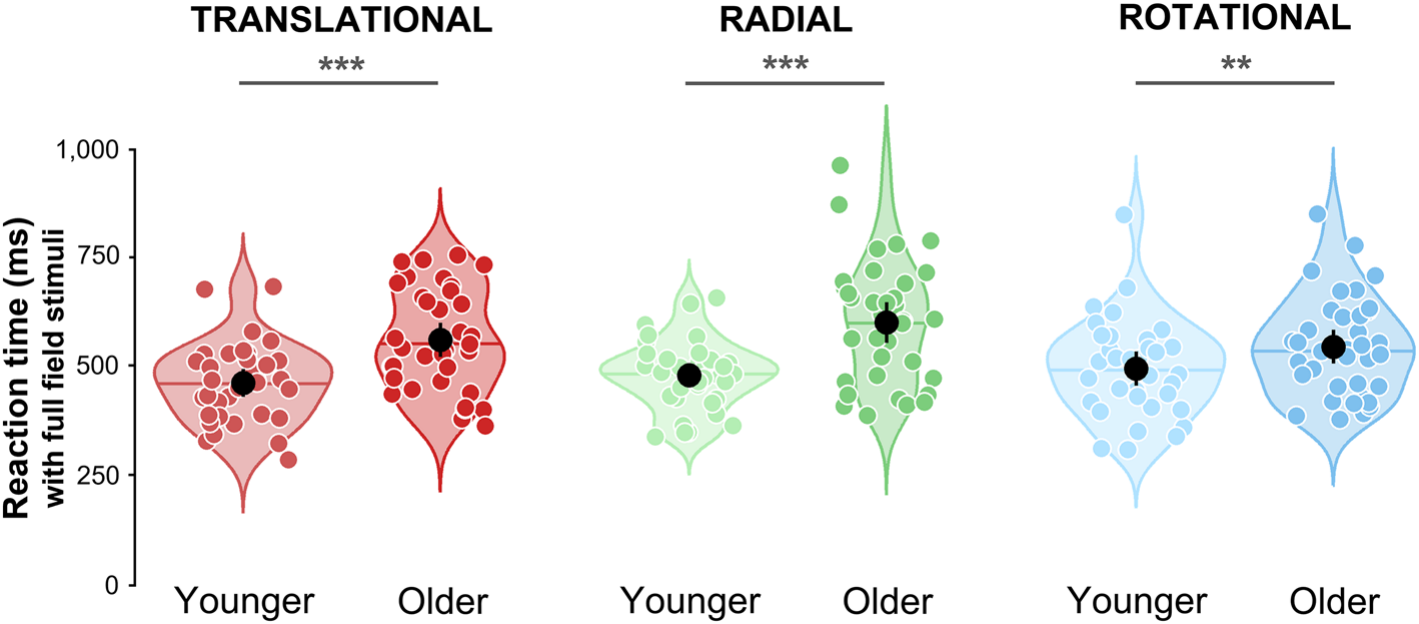
Reaction times distributions measured for full-field translational (red), radial (green) and rotational (blue) patterns. See Fig. 2 for more details.

Next, we examine the reaction time distributions in the two groups, which are shown in Fig. 3. Distributions of the data collected in the peripheral vision condition (with a central scotoma) are provided in Supplementary Text S3 and Fig. S3.

Another two-way ANOVA was performed to examine reaction times (RT), with the group (younger or older participants) and the gender as between factor and the optic flow pattern (translational, rotational or radial) and the viewing condition (full-field or peripheral vision) as within factors. This ANOVA showed a significant group effect, F(1, 65) = 1.62, p < 0.001, η_p_^2^ = 0.204. Reaction times were on average 97 ms longer in older participants (mean RT = 478 ± 93 ms for younger participants and mean RT = 575 ± 129 ms for older participants, *d* = 0.857). A significant effect of the optic flow pattern was also found, F(2, 130) = 5.72, p = 0.006, η_p_^2^ = 0.092. Post-hocs t-tests (with Welch’s adjustment) corrected for multiple comparisons (Bonferroni) demonstrated that reaction times were shorter for translational than for radial patterns (mean RT = 514 ± 119 ms for translational patterns, mean RT = 540 ± 130 ms for radial patterns, p < 0.001, *d* = 0.205). No difference was found between translational and rotational (mean RT = 526 ± 118 ms for rotational patterns, p = 0.257, *d* = 0.109) or radial and rotational patterns (p = 0.501, *d* = 0.099). The ANOVA also led to a significant interaction between group and optic flow pattern, F(2, 130) = 1.07, p < 0.001, η_p_^2^ = 0.133. Post-hocs t-tests corrected for multiple comparisons showed that younger participants had shorter reaction times for translational (mean RT translational = 454 ± 87 ms for younger participants and mean RT = 573 ± 117 ms for older participants, t(135.48) = 6.73, p < 0.001, *d* = 1.144), rotational (mean RT rotational = 497 ± 109 ms for younger and mean RT = 555 ± 121 ms for older participants, (t(135.44) = 3.00, p = 0.003, *d* = 0.511) and radial patterns (mean RT radial = 481 ± 78 ms and mean RT = 596 ± 145 ms for older participants, t(123.11) = 5.67, p < 0.001, *d* = 0.960). Post-hoc pairwise t-tests also revealed that younger participants had shorter reaction times for translational than radial (p < 0.001, *d* = 0.335) and rotational patterns (p < 0.001, *d* = 0.396). No differences were found between radial and rotational patterns (p = 0.38, *d* = 0.105). Older participants responded significantly faster for rotational than radial patterns (p = 0.005, *d* = 0.288), and no differences were measured between radial and translational (p = 0.243, *d* = 0.143) or rotational and translational patterns (p = 0.157, *d* = 0.158). The ANOVA did not lead to a significant effect of the gender (F(1, 65) = 1.43, p = 0.235, η_p_^2^ = 0.001).

The ANOVA showed that reaction times were comparable in the two viewing conditions (F(1, 65) = 0.25, p = 0.617, η_p_^2^ = 0.001). No interaction between the viewing condition and the other variables was found (p > 0.05).

### Speed effects on the processing of optic flow patterns in younger and older participants

**Figure 4:**
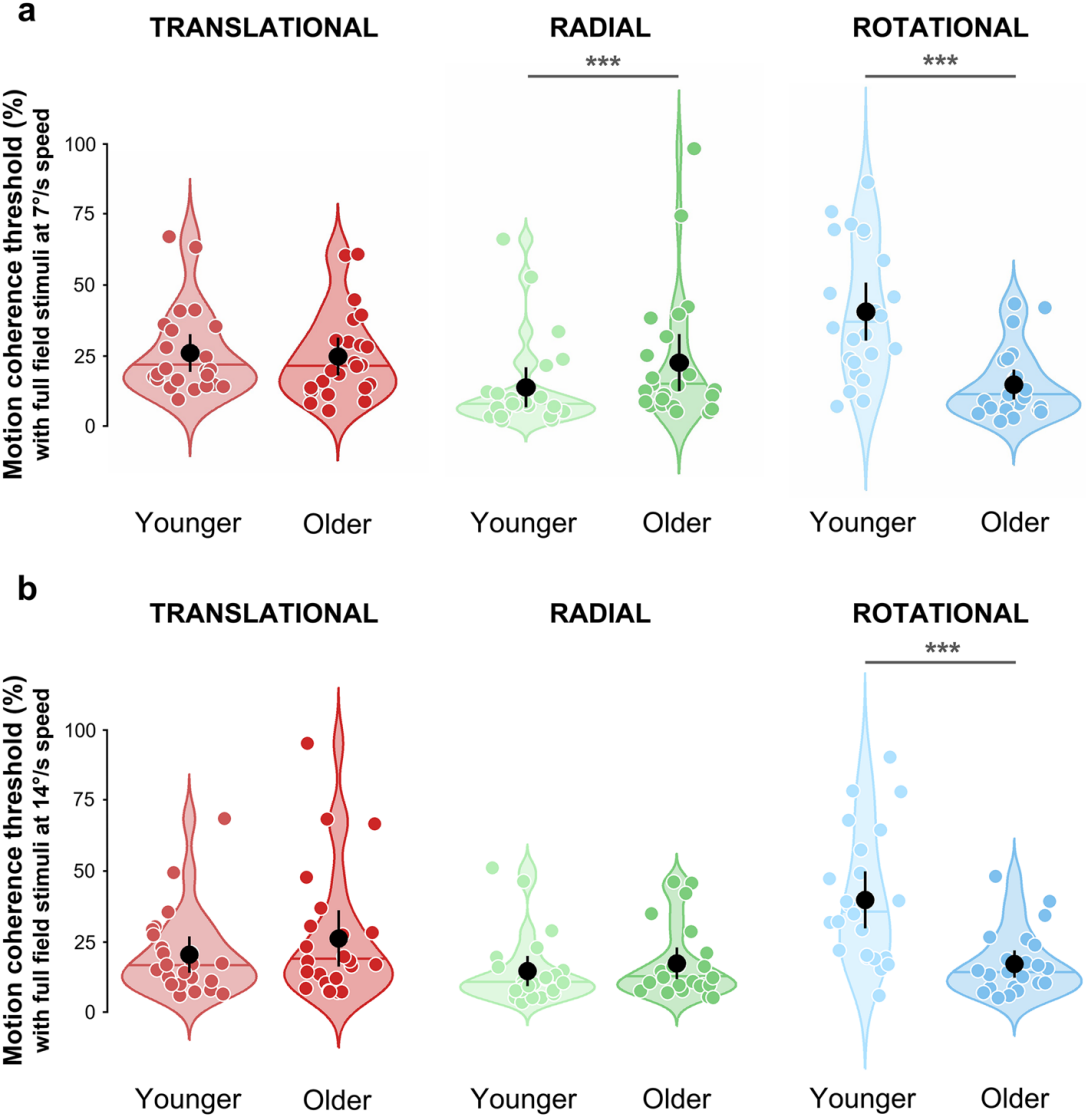
Distributions of motion coherence thresholds estimated for full-field translational (red), radial (green), and rotational (blue) optic flow patterns moving at 7°/s (**a**) and 14°/s (**b**). See Fig. 2 for more details.

Additional recordings were performed with an average dot speed of 14°/s in a subgroup of 23 younger (16 women, mean age = 23.3 ± 2.61) and 23 older (13 women, mean age = 73.3 ± 4.53) adult participants. Figure 4 shows the distributions of motion coherence thresholds measured in the two groups for the three components of optic flow with speeds of 7°/s (Fig. 4a) and 14°/s (Fig. 4b). Distributions of the data collected in the second viewing condition (peripheral vision) are provided in Supplementary Fig. S4.

We ran an ANOVA with the group (younger or older participants) and the gender (women or men) as between factors and the optic flow pattern (translational, rotational or radial), the viewing condition (full-field or peripheral vision) and the speed (7°/s or 14°/s) as within factors. The pattern of results was close to the one described above. Motion coherence thresholds were globally higher in the presence of a simulated scotoma, F(1, 42) = 6.06, p = 0.018, η_p_^2^ = 0.128 (mean full-field = 23.5 ± 19.3%, mean peripheral vision = 24.7 ± 18.7%, *d* = 0.124). As reported for the whole group of participants, thresholds in the aged population were significantly higher for the radial patterns and lower for the rotational patterns (respectively *d* = 0.458 and *d* = 1.292, see detailed results in Supplementary Text S4). Contrary to the previous results, no significant interaction between the gender and the pattern was found (F(2, 84) = 1.51, p = 0.226, η_p_^2^ = 0.004). This ANOVA also showed a significant effect of the speed, F(1, 42) = 5.60, p = 0.023, η_p_^2^ = 0.164 with higher coherence thresholds at the lowest speed (mean 7°/s = 24.9 ± 18.3%, mean 14°/s = 23.3 ± 19.6%, *d* = 0.156). Additionally, the interaction between the speed and the group was significant (F(1, 42) = 3.55, p = 0.033, η_p_^2^ = 0.028). Post hoc pairwise t-tests showed that coherence thresholds were similar at the two different speeds in the younger participants group (mean 7°/s = 27.3 ± 20.7%, mean 14°/s = 26.1 ± 21.2%, p = 0.053, *d* = 0.095), but older participants had significantly lower thresholds at higher speed (mean 7°/s = 22.6 ± 16.4%, mean 14°/s = 20.5 ± 17.4%, p = 0.007, *d* = 0.229). The interaction between the speed and the optic flow pattern was significant as well (F(2, 84) = 3.69, p = 0.035, η_p_^2^ = 0.100). Post hoc t-tests notably indicate that thresholds were lower at higher speed for the translational patterns (*d* = 0.332), but no difference was found for the two other patterns of motion (see Supplementary Text S4 for more detailed results). Finally, the ANOVA showed a significant interaction between the group, the optic flow pattern and the speed, F(2, 84) = 5,61, p = 0.007 , η_p_^2^ = 0.096. As a post-hoc analysis, we ran two additional ANOVAs on this dataset, the first one using only the data with an average speed of 7°/s and the second one using only the data with an average speed of 14°/s. These analyses demonstrated that for the slowest speed (7°/s), we replicated the results described above (see Section "Age effects on the processing of optic flow patterns in younger and older participants") on a smaller group of participants (here as well, older participants had higher thresholds for radial patterns but lower thresholds for rotational patterns than the younger group). With the 14°/s data, the ANOVA also led to a significant effect of the optic flow pattern and to an interaction between the group and the optic flow pattern (respectively F(2, 84) = 18.65, p < 0.001, η_p_^2^ = 0.317 and F(2, 84) = 15.56, p < 0.001, η_p_^2^ = 0.281). However, post-hoc analysis showed that if the effect for the rotational patterns remained unchanged (higher thresholds in younger participants, t(89.96) = 6.13, p < 0.001, *d* = 1.278), the difference between younger and older participants for the radial patterns was not observed anymore at a higher motion speed (t(89.65) = 0.99, p = 0.323, *d* = 0.207, see Fig. 4).

Reaction time distributions in the two subgroups were also analyzed using an ANOVA with the group (younger or older participants) and the gender (women or men) as between factors and the optic flow pattern (translational, rotational or radial), the viewing condition (full-field or peripheral vision) and the speed (7°/s or 14°/s) as within factors. Results were similar to the ones on the larger group, with higher reaction times for older participants (see Supplementary Text S5 and Supplementary Fig. S5 for more details), except for the effect of the gender which was significant on this new dataset, F(1, 41) = 4.22, p = 0.046, η_p_^2^ = 0.044. Women had longer RT than men (mean women = 548 ± 125 ms, mean men = 511 ± 125 ms, *d* = 0.322).

Altogether, these results suggest that motion coherence thresholds decrease with higher motion speed, especially in older participants. The processing of radial patterns in older adults could notably be impaired at slow but not at high speeds.

### Control for potential biases in the measurements

**Figure 5:**
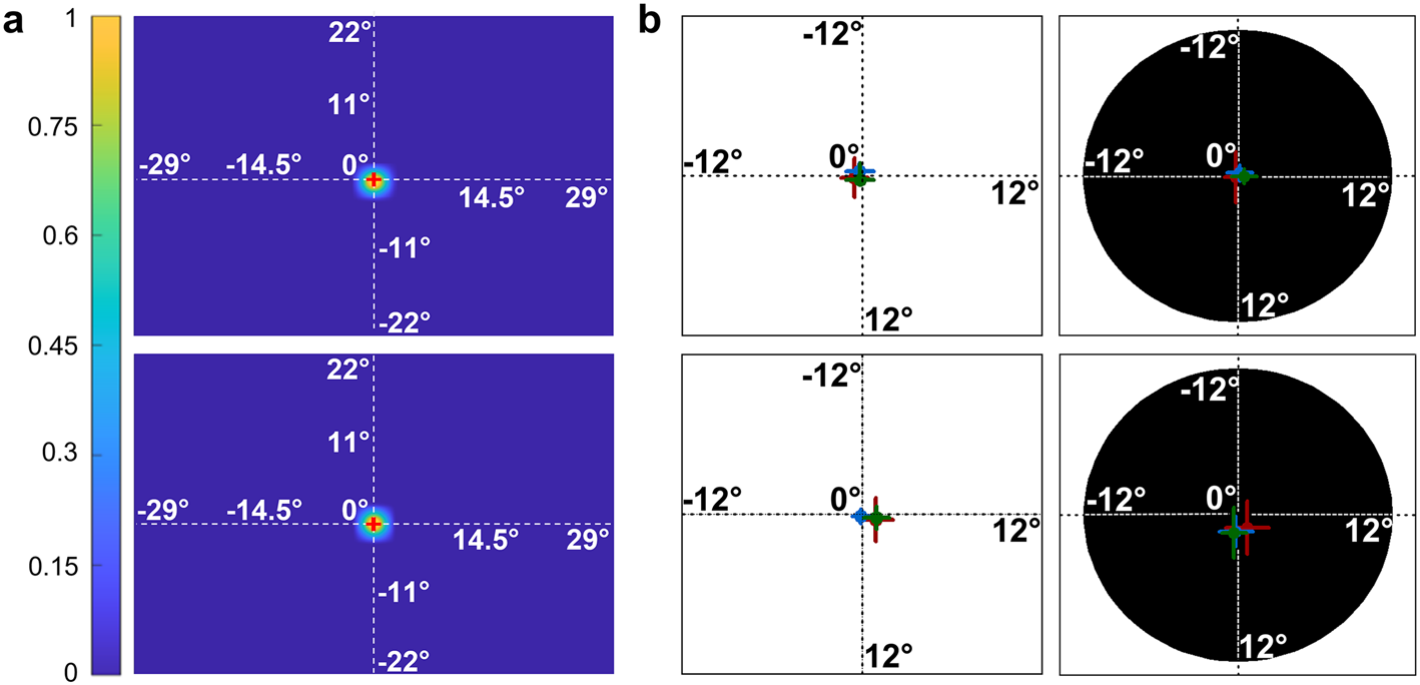
Ocular fixation. (**a**) Normalized fixation durations across conditions in two representative participants, one younger (upper row) and one older (lower row) adult. The point of ocular fixation is given by the central red cross. (**b**) Average eye positions relative to the fixation cross in the groups of younger (n = 42, upper row) and older (n = 42, lower row) participants for the two viewing conditions (full-field in the left column and peripheral vision with an artificial scotoma on the right column). The red, green and blue discs respectively represent these positions for translational, radial and rotational optic flow patterns. The horizontal and vertical segments provide the 95% confidence intervals on the x and y axes. Although the stimulated portion of the visual fields covered 58.1° by 43.7°, we only represented here the near surround of the fixation cross (± 12°) to improve the visibility of the data.

In this section, we test for potential biases in our measurements. Figure 5 shows the normalized ocular fixation durations (see the "Methods" section) in two typical participants (one younger and one older adults, Fig. 5a) and across all the participants in the younger and older adult groups (Fig. 5b). We can observe that gaze fixation was very stable in the two groups and for all the conditions. These analyses show that our results are not corrupted by instability in ocular fixation.

For each optic flow pattern, we examined whether behavioral responses were biased toward one direction (toward leftward or rightward motion for translational patterns, toward contraction or expansion for radial patterns, or toward counterclockwise or clockwise motions for rotational patterns). The comparison between proportion of correct responses in each direction for each pattern did not lead to significant bias in any experimental conditions and for neither of the two groups. Results are shown in Supplementary Fig. S6.

## Discussion

The aim of this study was to characterize optic flow processing in older adults. This is important because this population mostly relies on visual cues for navigation^10^. We used an adaptive Bayesian approach to estimate motion coherence thresholds for translational, rotational and radial patterns in a group of older (> 70, n = 42) adults and in a group of younger controls (18–30, n = 42). We found different effects for the different patterns.

The translational pattern is by far the most explored in the aging literature. Studies based on RDKs generally reported that motion direction discrimination thresholds are increased in older adults for this pattern^33,34^, notably in participants over 70^18,35^. Using correlational designs, several research groups found a constant diminution of thresholds with age^14–16^. However, other studies did not observe any effect of age for direction discrimination tasks based on planar motion. For example, Kavcic et al.^27^ found that thresholds were similar in groups of younger and older adults (see also Tetewsky & Duffy^36^ or Hutchinson et al.^37^ for similar results). Several factors have been proposed to explain these discrepancies^4,31^, such as the speed, the size or the contrast of the stimulus. In particular, Snowden and Kavanagh^21^ found that older adults had higher thresholds than younger participants only for velocities below 2°/s. In our study, we did not observe any age effect on the processing of translational motion. It is possible that the moderate to high speeds that we used (7°/s and 14°/s) did not permit us to measure an effect, in line with previous findings (see e.g. Allen et al.^19^ for dots at high contrast moving at 5.6°/s). This hypothesis is supported by our statistical analyses for which we found that thresholds in older participants were significantly better at higher velocities whereas performances remain stable across speeds for younger adults. We found that thresholds for translational patterns were higher in women than in men for slower patterns (7°/s). This effect was limited to planar motion as we did not observe gender differences for the processing or radial and rotational patterns (nor interactions implying gender). In the aging literature, numerous studies reported that motion discrimination thresholds were higher in older women than in older men^22,38^. More recent data^21,39^ showed that these gender differences can also be observed in younger participants and may therefore be present at all ages^4^. Our data are in line with these results.

The effects of age on the processing of radial optic flow patterns were less explored than those on the processing of planar motion and remain unclear. Warren et al.^23^ originally reported that heading thresholds were slightly increased in older participants. In later psychophysical studies, some authors found that heading estimation from radial flow was significantly impaired in older adults^24–27^ while others found that it was not^14,22^. Other studies led to intermediate results. For example, O’Brien et al.^28^ found an age effect on heading perception when participants had to discriminate between left versus right fields of expansion (FoE) but not when they had to discriminate between inward and outward flow fields (see also Mapstone et al.^29^ where effects were observed for self-motion simulated by moving objects but not by clouds of dots). Allen et al.^19^ found that younger participants had a better heading perception from radial flow than older adults but mainly for low contrast stimuli. These studies differed in many aspects (in the nature of the tasks, size of the stimuli and local motion properties) and it is difficult to extract a single parameter that could explain the divergences between the reported results. In our study, we found that thresholds for radial patterns were significantly higher in the older participants but only in our lower speed condition (7°/s on average). This effect was not observed anymore for faster radial flow (14°/s on average). Importantly, this difference cannot be attributed to the lower number of subjects who underwent the experiment with the higher speed (n = 46 versus n = 84) because age effects were still observed for the lower speed on this subsample. Our results therefore suggest that the processing of radial patterns in older adults could be impaired at slow but not at high speeds, as it was proposed for the processing of translational patterns^21^. If velocity can probably not fully account for the discrepancies between the previous studies (for example, both Lich and Bremmer^24^ and Falkenberg & Bex^25^ found an age effect on heading perception although the speeds of their stimuli differed by a ratio of about 14), it is a parameter that was not systematically explored and its impact on heading perception in older adults should be further characterized in future research works.

Rotational patterns are very rarely used in studies on optic flow processing in humans (see however Strong et al.^13^ or Guénot et al.^32^) whereas this pattern is quite frequent in everyday life because of the head rotation during walking. Rather surprisingly, we found that older participants had lower thresholds than younger ones when they had to discriminate between noisy dot patterns rotating either clockwise or counterclockwise. This effect was large and robust (it was observed in all our conditions). To our knowledge, there is only one study which explored how age affects the perception of rotational patterns^19^. The authors reported that older participants had lower thresholds when they discriminated between clockwise and counterclockwise rotations but mostly at low contrast values. In this case, stimuli were restricted to a 12° of diameter circular window, which is much smaller than the 58.1° × 43.7° used in our study. More importantly, rotational (and also radial) patterns in this study did not include velocity gradients (i.e., dots moved at a constant speed of 5.6°/s) and thus reflected global motion rather than optic flow. These important differences are likely to explain the discrepancies between the results obtained in the two studies. In our data, the effect observed for rotational patterns was mainly driven by the higher thresholds measured in the younger population (see Fig. 2). It is not the first time that poorer performances in younger adults are reported in a motion discrimination task. Indeed, Betts et al.^40^ found that younger participants were worse at discriminating the motion direction of a sinewave grating when it was presented at high contrast and covered a large portion of the visual field, as it is the case in our experiment. The authors attributed this observation to spatial suppression, a neural mechanism that promotes rapid figure-ground segmentation of moving objects^41^ (and might thus facilitate flow parsing) through surround inhibition signals in neurons of motion selective areas^42,43^. These inhibitory signals are mediated by feedback from higher-level cortical regions^44^ and could be reduced with age, due to altered GABAergic functioning^45^. It is possible that our results also reflect a form of spatial suppression because in primates, rotational patterns are processed in cortical areas where center-surround antagonistic mechanisms were reported (see e.g. Born^46^, Raiguel et al.^47^ or Eifuku & Wurtz^48^ for electrophysiological recordings in areas MT and MST of macaques or Er et al.^49^ for fMRI data in the human hMT + complex). Further investigations (e.g., using a parametric modulation of the stimulus size as in Hutchinson et al.^20^) will be needed to clarify this point and also why threshold improvements in older adults were only observed for rotational patterns. It is possible that spatial suppression effects also exist for translational and radial patterns but were not detected in our study because they are counterbalanced by other mechanisms (e.g., a weaker selectivity of the associated neural populations in older adults). Altogether, our study points toward different age effects on the processing of the three optic flow patterns, which is consistent with the fact that they are processed by distinct neural populations along the visual system hierarchy^12,13^.

In our experiments, motion discrimination thresholds were higher when an artificial scotoma occluded central vision (when only peripheral vision was stimulated, see Supplementary Text 1). In a previous study based on the same experimental protocol^32^, we had found that performances were unaffected when central vision was masked. This discrepancy might be explained by the fact that the effect of the artificial scotoma is small and only detectable with a large group of participants (n = 84 in this study versus n = 12 in the previous one). To verify this hypothesis, we ran additional statistical analyses on a subgroup of 12 subjects from the present study and did not observe any effect in this case. In the literature, if optic flow is believed to be predominantly processed by peripheral vision^50,51^, some contributions of central vision were also reported^52^. Altogether, our data points toward a moderate but nonetheless significant implication of central vision on optic flow processing. A previous study reported^30^ that planar motion perception in older adults is more impaired in central vision. Our results are not totally in line with this observation as the effects of the viewing condition in our data were independent of the age group for all the optic flow patterns. They thus suggest that age effects on motion perception remain the same across the visual field. Additional recordings using artificial scotoma of different sizes and also stimuli restricted to central vision will be needed to properly test this hypothesis.

One limitation of our study is that we only considered two distinct classes of age (18–30 and 70–90 for the younger and older adult participants) as opposed to a continuum that could have permitted to more finely characterize how changes in optic flow processing evolve across adulthood (see Billino & Pilz^4^). Such sampling could notably help to clarify whether perceptual changes are progressive or rather appear at a certain age. Another potential limitation is that our optic flow patterns were based on random dot kinematograms (RDKS). If these stimuli permit to precisely isolate motion patterns and to control their coherence level, they remain very abstract and additional experiments using more ecological conditions (e.g., including optic flow patterns embedded in rich stimuli) will be needed to determine whether the results of the present study remain unchanged in an everyday life context.

Optic flow can elicit eye movements^53^. To control that the motion discrimination thresholds estimated in our two groups of participants were not affected by these eye movements or unstable ocular fixations, we instructed our participants to gaze on a central cross while their ocular fixation was recorded with an eye-tracker. Our analyses revealed that both the two groups had very stable gazes. In particular, we did not find evidence for eye stability differences between the younger and older adults, in line with a previous study which reported that the accuracy of eye movements did not differ across age^18^ (but see Munoz et al.^54^ or Knox et al.^55^ in the context of saccade latency and smooth pursuit). It has to be noted that in real life situations and notably during locomotion, the eyes (and also the head) are moving. Based on recordings performed in a group of younger participants walking through real-world natural environments, a recent study proposed that eye movements could serve for the stabilization of optic flow projections on the retinas^56^ and thereby facilitate the adaptation of walking speed and direction. It would be interesting to explore whether these effects are deteriorated for aged participants in whom deficits have been observed during walking^57,58^.

In conclusion, we found that age effects on the processing of the three components of optic flow (translational, radial and rotational patterns) differ. Motion discrimination thresholds were increased in older adults for radial patterns at lower speeds but reduced for rotational patterns. In line with previous studies^4,59^, these findings support the idea that aging does not lead to a general decline in visual perception but rather has specific effects not only on the perception of different types of motion (local, global and optic flow) but also on more specialized motion processing such as the integration of the different optic flow patterns. They have important implications for the development of assisting devices for older adults, notably in the context of locomotion.

## Methods

### Participants

Forty-two older participants aged 70–90 (18 females, mean age: 73.52 ± 4.24) and forty-two younger participants aged 18–30 (25 females, mean age: 23.88 ± 3.01) were included in the study. None of these participants had any known ocular disease nor history of neurological disorders. They all had a corrected visual acuity over 7/10 in at least one eye. Visual acuity was measured monocularly in each eye using the Sloan letters of the Freiburg Visual Test^60^. Participants were recruited via advertisements in local journals. The research was conducted at the Centre de Recherche et Cognition (Toulouse, France) and the experimental protocol was approved by a national institutional ethical committee before the beginning of the study (CPP, Comité de Protection des Personnes, protocoles 13018–14/04/2014 and 2020-A02441-38). All research was performed in accordance with relevant guidelines and regulations. Informed written consent was obtained from all participants prior to the experiments.

### Optic flow stimuli

Motion stimuli were adapted from the experimental protocol of a previous electrophysiological study^61^. They consisted in translational, rotational or radial optic flow patterns defined from random-dot kinematograms (RDKs) (see Fig. 1) generated with Matlab (R2017a) using the Psychophysics toolbox. They were presented on a large convex screen (58.1° × 43.7° of visual angle, refresh rate: 60 Hz, resolution: 1400 × 1050 pixels) at a viewing distance of 180 cm. The experiments took place in a dark room where the screen was the only light source. The RDKs contained bright non-overlapping dots (diameter: 0.2°) moving on a homogenous dark background with a high contrast (100%). They had a density of 0.3945 dots per degree of visual angle. Each dot had a limited lifetime of 200 ms (12 frames), during which it moved at a constant speed along a straight direction. At the end of this lifetime, the dot was randomly reassigned to a new spatial position within the screen and given a trajectory and speed corresponding to this new position. To avoid a coherent flickering of the stimulus every 200 ms, each dot initial age was randomly picked between 0 and 166 ms (11 frames) at the beginning of each trial. When a dot reached the border of the display screen, it was immediately relocated at a random position. These processes permit to equalize mean luminance and dot density across the screen during the whole experiment. We used a velocity of 7°/s for the translational condition. This value corresponds to the average preferred speed of neurons in macaque MT. Both the radial and rotational optic flow patterns had identical speed distributions. In these conditions, the speed of a dot was a function of its eccentricity *Ecc* and was given by S × Ecc. S was chosen to equalize the average speed in the radial and rotational conditions with the speed in the translational condition, in order to obtain comparable thresholds between the three different types of optic flow patterns. For these two conditions, the field of expansion (FoE) was placed at the center of the screen. Because dot size, density and speed were equalized between the translational, radial and rotational optic flow patterns and because the trajectories contained no curvature or acceleration, the only difference between these three conditions was the optic flow pattern.

To determine whether speed had an influence on our results, we also used stimuli with dots moving at 14°/s on average in a subset of our participants (23 younger and 23 older adults).

### Experimental design

During the experiments, participants sat in a chair whose height was adapted in order to equalize the height of the eyes with the center of the screen. Their head was placed on a head-support device clamped on top of a table and equipped with both chin and forehead supports. The chair and head-support devices were positioned so as to ensure a fine alignment between the participants’ head and trunk axes. Participants had to keep this position as constant as possible. The task was performed monocularly with the eye having the best visual acuity or with the dominant eye when the visual acuity was equal in both eyes. The other eye was patched. Participants had to gaze on a central fixation cross during the whole experiment.

A two-alternative forced-choice task (2-AFC) was used to estimate motion discrimination thresholds for each of the three optic flow patterns (translational, rotational or radial). Stimuli were presented in blocks of 64 trials. Each block contained only one optic flow pattern and lasted about 3 min. Each trial started with the presentation of the stimulus for 200 ms. Participants had to report the motion direction of the stimuli: leftward versus rightward for translational, clockwise versus counterclockwise for rotational patterns, and inward versus outward for radial. They were instructed to respond as quickly as possible while maximizing their performances. Responses reported after 2 s were considered as incorrect. After each trial, an auditory feedback specified whether the chosen direction was correct or not. Because our stimuli were presented for a very short duration, it is unlikely that they elicited vection.

During each block, we manipulated motion coherency (i.e., the percentage of dots moving along the same direction while the other dots had random directions) and estimated the thresholds corresponding to 80% of correct detection using an adaptive Bayesian approach (QUEST, see below). For each optic flow pattern, the first block was considered as a training and not included in the analyses. Our two groups of participants completed the study in two viewing conditions. In the first one, stimuli were presented full screen, while in the second one, only peripheral vision was stimulated, as a simulated scotoma (i.e., a black disk) of 10° of radius masked the center of the screen. This second condition was used to evaluate the contribution of central vision in our task. Thresholds were estimated for the three optic flow patterns (translational, rotational and radial) in these two viewing conditions (full-field stimuli and peripheral stimuli). Participants completed three blocks of each condition. Breaks were included (3 to 4 min on average) between blocks to reduce fatigue in older participants. Blocks and condition sequences were randomized to minimize possible learning effects on the results. The whole experiment was completed in one session of about an hour and a half.

A sub-group of 23 younger (16 women, mean age = 23.3 ± 2.61) and 23 older adults participants (13 women, mean age = 73.3 ± 4.53) also performed the same experiments (three blocks of optic flow patterns, full-field and peripheral vision) with an average dot speed of 14°/s.

### Robust estimation of motion coherence thresholds using QUEST

Because long psychophysical measurements are difficult to perform in aged populations, we used the QUEST adaptive procedure to obtain rapid, efficient and robust estimations of motion coherence thresholds. QUEST is a Bayesian method that assumes that the psychophysics function underlying the participant performance follows a Weibull distribution. During a block, the estimated parameters of this function were updated after each trial on the basis of the participant’s response. Coherence values corresponded to the current maximum likelihood estimate of the threshold. We fixed the maximum number of trials at 64 as it was previously shown that this value leads to robust thresholds in most circumstances^62^. We used an initial threshold value of 58 percent based on previous recordings using the same stimuli^32^. The robustness and reproducibility of our psychophysical protocol was also validated from tests-retests performed on four participants. Results showed that the estimated thresholds were very stable across both blocks and sessions.

### Statistical analyses

Each participant performed three blocks of each pattern (translational, radial and rotational) in the two viewing conditions (full-field or peripheral vision). The three corresponding motion coherence thresholds were subsequently averaged together.

In our statistical analyses, we first evaluated age effects on the processing of the different optic flow patterns. This was realized using two multi-factorial ANOVAs (one on motion discrimination thresholds, the other one on reaction times) performed on the data collected at 7°/s in all the participants (42 younger and 42 older adults). In this case, our factors of interest were: age group (younger and older adults), optic flow pattern (translational, radial and rotational), viewing condition (full-field or peripheral vision) and gender (men or women). The associated effects are reported in Section "Age effects on the processing of optic flow patterns in younger and older participants".

In a second step, we characterized speed effects on the processing of optic flow patterns using the data collected in the subgroup of participants who underwent the experiments at both speeds (23 younger and 23 older adults, see above). This was realized with two additional multi-factorial ANOVAs (one for motion discrimination thresholds and the other one for reaction times) that included speed (7°/s or 14°/s) as a factor of interest in addition to those reported above. The associated results are reported in Section "Speed effects on the processing of optic flow patterns in younger and older participants".

Because distributions of discrimination thresholds are generally non gaussian and right-skewed, all the data were normalized using a log10 transformation prior to statistical analyses^63^. In addition, a Greenhouse-Geisser^64^ correction was applied when appropriate (i.e., when the Mauchly’s sphericity was statistically significant). Significant effects of the ANOVAs were explored with post-hoc t-tests and paired t-tests. To facilitate the comparison with previous studies, the means and standard deviations (SD) reported in the text and in the figures correspond to the coherence level in percentage (i.e., before the log10 transformation).

### Measures of ocular fixation

To control that our results were not corrupted by instability in ocular fixation, for each of the participants, eye position was measured using an eye-tracker (EyeLink 1000, sampling frequency: 1 kHz) placed at 35 cm in front of them. We estimated the average eye position during the 200 ms of the stimuli. Trials with blinks were removed from the analyses.

### Approval statement

The research was conducted at the Centre de Recherche et Cognition (Toulouse, France). All methods were carried out in accordance with relevant guidelines and regulations. The experimental protocol was approved by a national ethical committee before the beginning of the study (CPP, Comité de Protection des Personnes, protocoles 13,018–14/04/2014 and 2020-A02441-38).

### Consent statement

Informed consent was obtained from all participants prior to the experiments.

### Supplementary Information

Supplementary Information.

## Data Availability

The datasets used and analysed during the current study are available from the corresponding author on reasonable request.
