# Supplementary material for: Processing of translational, radial and rotational optic flow in older adults
Source: Sci Rep. 2023 Sep 15;13:15312. doi: 10.1038/s41598-023-42479-2 (PMC10504320; doi:10.1038/s41598-023-42479-2)
Supplement: Supplementary file 1 — Supplementary Information. [file 41598_2023_42479_MOESM1_ESM.docx]

**Processing of translational, radial and rotational optic flow in older adults**

**Jade Guénot* ^1^, Yves Trotter^1^, Angélique Delaval^1^, Robin Baurès^1^, Vincent Soler ^2^ and Benoit R. Cottereau ^1^**

^1^ Brain and Cognition Research Center – Université Toulouse III - Paul Sabatier – France

^2^ Hôpital Purpan, Unité de rétine – CHU Toulouse – France

***** : Corresponding author.

**Supplementary materials**

**Supplementary Text S1: Age effect on motion coherence thresholds obtained with peripheral (i.e. masked with a central scotoma) optic flow stimuli presented at 7°/s**

The ANOVA showed a significant effect of the viewing condition (full-field or peripheral vision), F(1, 80) = 7.85, p = .006, η_p_² = 0.079 (mean full-field = 24.9 ± 19.6 %, mean peripheral vision = 25.1 ± 17.4 %). No significant interaction between the viewing condition and the pattern or this variable and the group was established (respectively F(2, 160) = 0.59, p = .558, η_p_² = 0.007 and F(1, 80) = 0.91, p = .344, η_p_² = 0.012). Therefore, even though the presence of a scotoma slightly increases motion coherence thresholds in all participants, the pattern of results obtained in the two viewing conditions when comparing the performances of the two groups are similar, as shown in Figure 2 (with full visual field) and Figure S1 (with a central scotoma). Older participants had higher motion coherence thresholds for the radial pattern and lower thresholds for the rotational pattern than the young participants, and this regardless of the viewing condition.


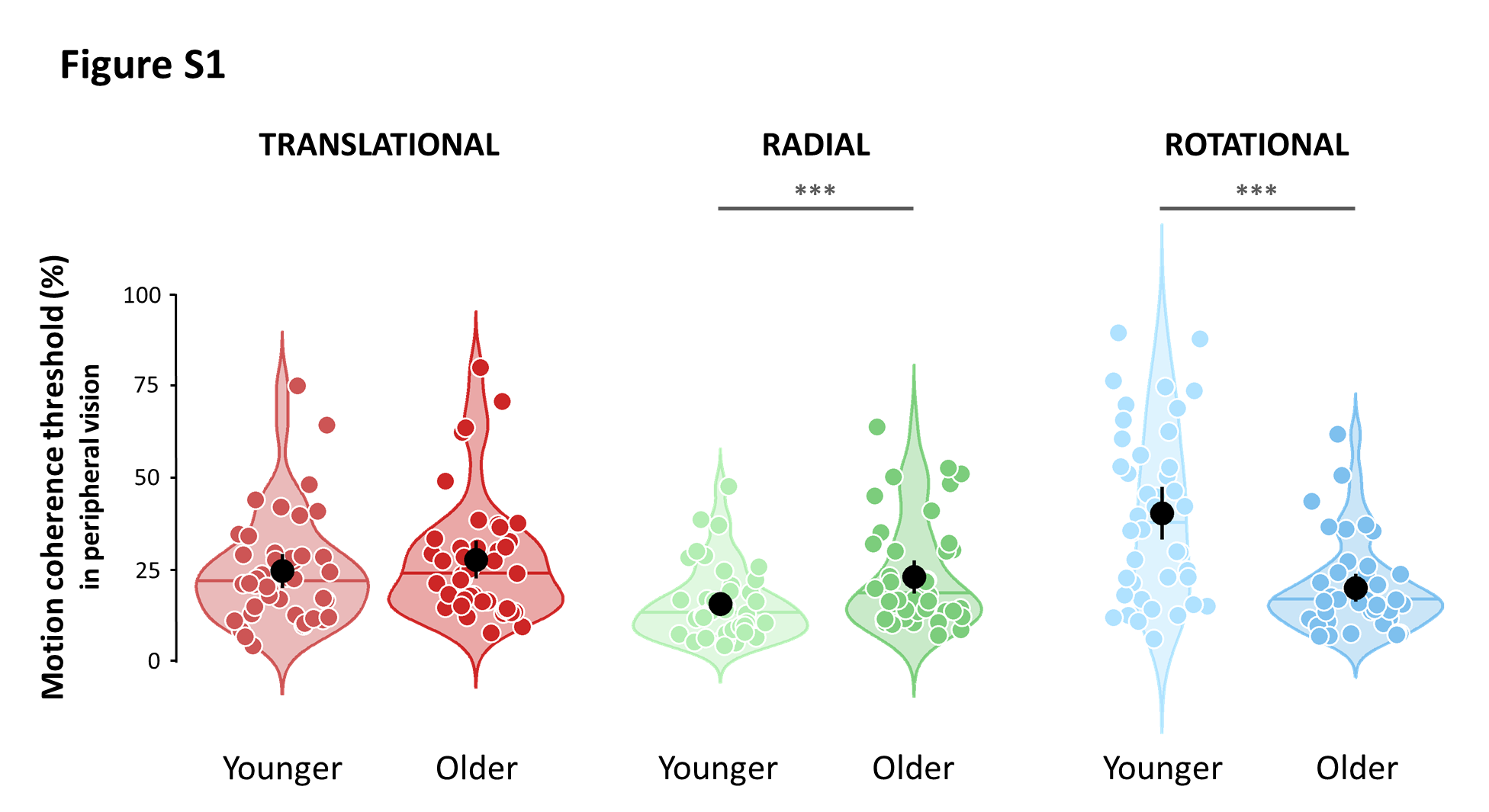


***Figure S1.*** *Distributions of the motion coherence thresholds estimated for translational (red), radial (green), and rotational (blue) optic flow patterns, for the young (age: 18-30, n = 42, light colors) and old (age: 70-90, n = 42, dark colors) adults groups when central vision was masked. Black dots represent group-level means. Error bars represent the associated 95 percent confidence intervals. Horizontal colored lines represent group-level medians. Circles provide the individual data points of the distributions. Note that they were slightly offset horizontally to improve their visibility. Stars indicate significantly different distributions (***, p < .001).*

**Supplementary Text S2: Gender effects**

In the aging literature, it has been reported that motion discrimination thresholds were higher in older women than in older men^40,22^. More recent data^21,41^ showed that these gender differences can also be observed in younger participants and may therefore be present at all ages^4^. Our data are in line with these results.

No gender effect was found (F(1, 80) = 2.21, p = .141, η_p_² = 0.030) but the interaction between gender and optic flow pattern was significant (F(2, 160) = 3.46, p = .034, η_p_² = 0.031). Post-hoc t-tests notably showed that women had higher thresholds than men for translational patterns only, regardless of their group (mean women = 29.1 ± 15.8 %, mean men = 22.4 ± 16.3 %, t(81.92) = 3.27, p = .002, *d* = 0.696), and that motion coherence thresholds were not significantly different between the two genders for rotational (mean women = 31.6 ± 22.4 % mean men = 28.2 ± 20.7 %, p = .345, *d* = 0.201) and radial (mean women = 19.7 ± 15.5 %, mean men = 18.9 ± 15.7 %, p = .332, *d* = 0.210) patterns. Higher translational thresholds were also found for older women compared to older men, or young women compared to young men (respectively p = .002, *d* = 0.576 and p = 0.014, *d* = 0.209 see Figure S2). Moreover, post-hoc pairwise t-tests indicate that women had lower coherence thresholds for radial patterns than for rotational (p < .001, *d* = 0.674) or translational patterns (p < .001, *d* = 0.808). No difference was found in women between rotational and translational patterns (p = 1.0, *d* = 0.057). Similar results appeared in men, with lower thresholds for radial than rotational (p = .002, *d* = 0.492) and translational patterns (p = .019, *d* = 0.342), and no difference between rotational and translational patterns (p = .448, *d* = 0.200). Finally, we did not find any interaction between the gender and the group or the viewing condition (p > 0.05).


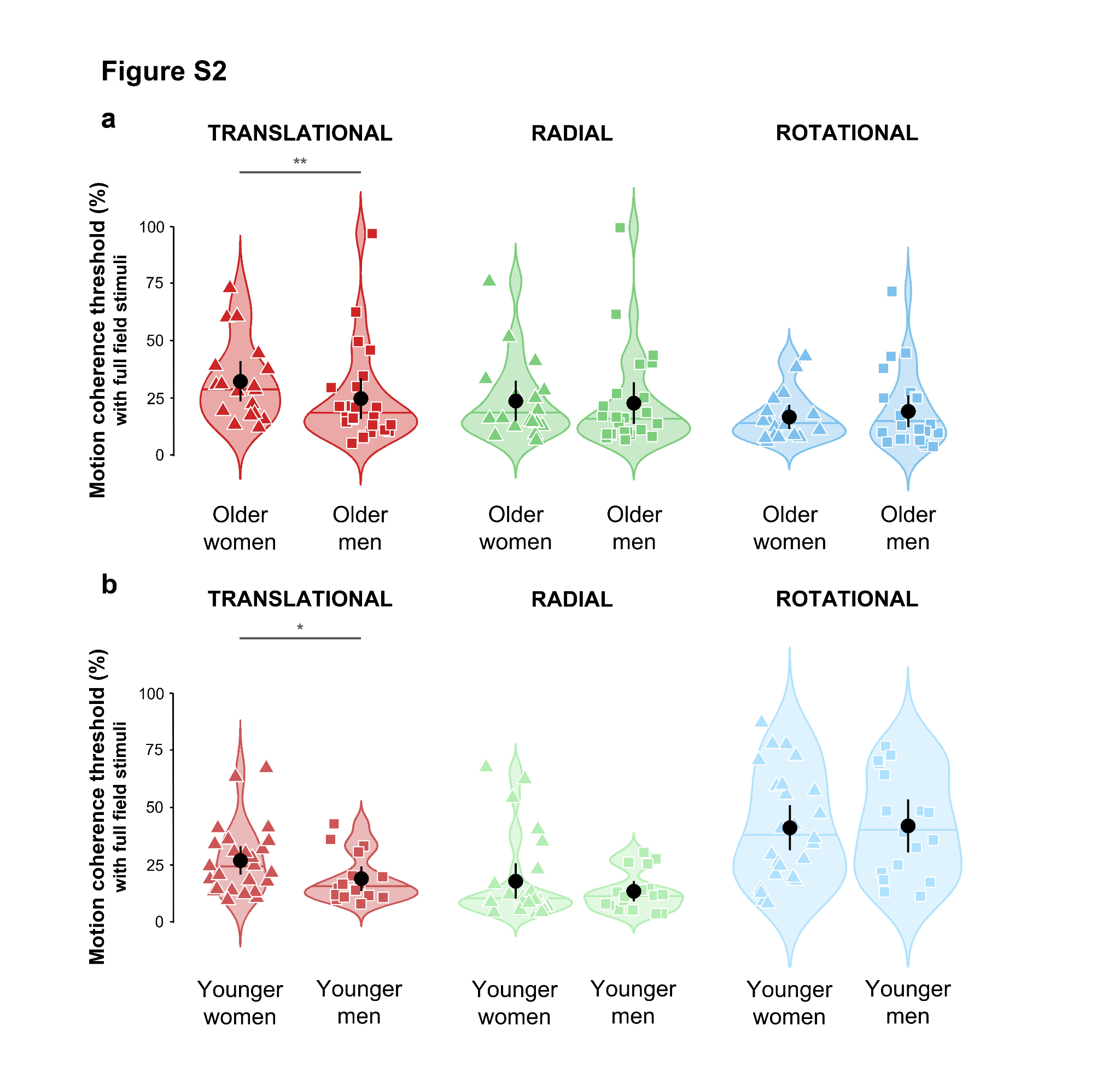


***Figure S2.*** *Distributions of the motion coherence thresholds estimated for translational (red), radial (green), and rotational (blue) optic flow patterns with full-field stimuli. Black dots represent group-level means of the (****a****) older women (triangles) and older men (squares) and (****b****) younger women (triangles) and younger men (squares). Error bars represent the associated 95 percent confidence intervals. Horizontal colored lines represent group-level medians. Circles provide the individual data points of the distributions. Note that they were slightly offset horizontally to improve their visibility. Stars indicate significantly different distributions (**, p < .01; *, p < .05). Similar results were obtained when central vision was masked.*

**Supplementary Text S3: Age effect on reaction times measured with peripheral (i.e. masked with a central scotoma) optic flow stimuli presented at 7°/s**

The ANOVA showed that reaction times were comparable in the two viewing conditions (F(1, 65) = 0.25, p = .617, η_p_² = 0.001), as shown in Figure. S3 and Figure 3. No interaction between the viewing condition and the other variables was found (p > .05).


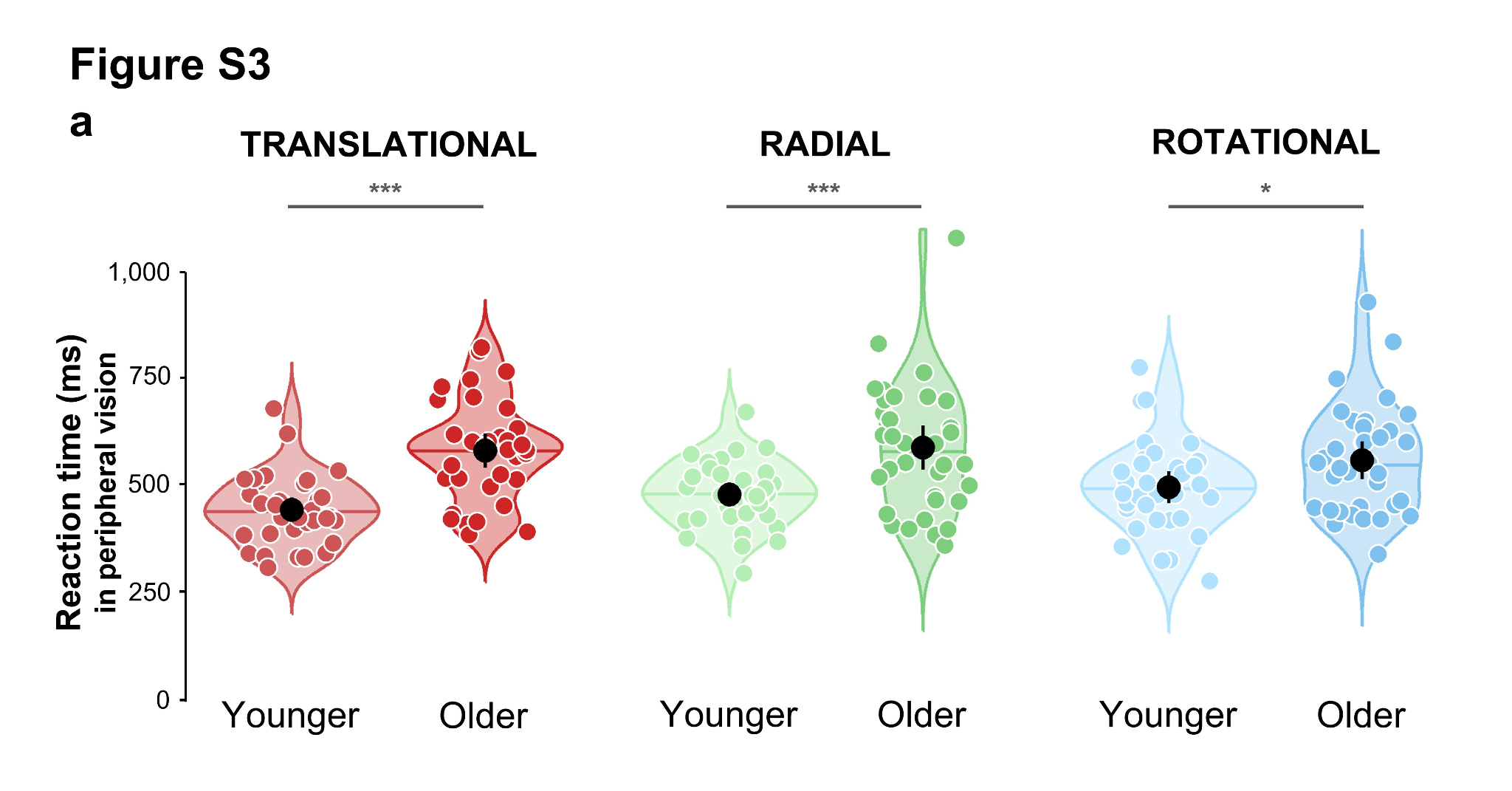


**Figure S3.** *Reaction times estimated for translational (red), radial (green), and rotational (blue) optic flow patterns when central vision was masked. See figure S1 for more details.*

**Supplementary Text S4: Effect of speed on motion coherence thresholds in younger and older adult participants**

An ANOVA with the group (young or older participants) and the gender (women or men) as between factors and the optic flow pattern (translational, rotational or radial), the viewing condition (full-field or peripheral vision) and the speed (7°/s our14°/s) as within factors was run. The effect of the viewing condition was significant, F(1, 42) = 6.06, p = .018, η_p_² = 0.128, with higher thresholds when the simulated scotoma was present (mean full-field = 23.5 ± 19.3 %, mean peripheral vision = 24.7 ± 18.7 %, *d* = 0.124). The results for the optic pattern and the interaction between group and pattern remained unchanged, respectively F(2, 84) = 19.57, p < .001, η_p_² = 0.335 and F(2, 84) = 25.29, p < .001, η_p_² = 0.378. Independently of the group, thresholds were lower for radial than for rotational (mean radial = 17.9 ± 15.4 %, mean rotational = 29.7 ± 21.5 %, p = .001, *d* = 0.705 and translational patterns (mean translational = 24.8 ± 17.7 %, p < .01, *d* = 0.588). Coherence thresholds did not differ between rotational and translational patterns (p = .431, *d* = 0.175). Similarly, older participant had higher thresholds for radial motion than the younger participants (mean young participant = 15.3 ± 13.7 %, mean old participants = 20.5 ± 16.5 %, t(181.96) = 3.11, p = .002, *d* = 0.458), lower thresholds for rotational patterns (mean young = 41.0 ± 23.2 %, mean old = 18.4 ± 11.8 %, t(181.99) = 8.76, p < .001, *d* = 1.292), and thresholds were equivalent in both groups for translational patterns (mean young = 23.7 ± 15.9 %, mean old = 25.8 ± 19.3 %, t(178.97) = 0.39, p = .697, *d* = 0.057).

The interaction between the speed and the optic flow pattern was significant as well (F(2, 84) = 3.69, p = .035, η_p_² = 0.100). Post-hoc t-tests indicate that thresholds were lower at higher speed for the translational pattern (mean translational 7°/s = 26.0 ± 14.8 %, mean translational 14°/s = 23.6 ± 20.1 %, t(171.42) = 2.29, p = .023, *d* = 0.332), but no difference at the different speeds were found for the rotational (mean rotational 7°/s = 29.8 ± 21.5 %, mean rotational 14°/s = 29.7 ± 21.7 %, t(181.99) = 0.02, p = .984, *d* = 0.003) or radial (mean radial 7°/s = 19.1 ± 16.6 %, mean radial 14°/s = 16.6 ± 14.0 %, t(181.97) = 1.29, p = .199, *d* = 0.190) patterns. Furthermore, at 7°/s and 14°/s, thresholds were lower for radial pattern than for rotational (p < .001, *d* = 0.608 and *d* = 0.803) and translational pattern (p < .001, *d* = 0.682 and *d* = 0.513), no difference was found between rotational and translational patterns (p = 1.0, *d* = 0.023 at 7°/s and p = .059, *d* = 0.316 at 14°/s).

***
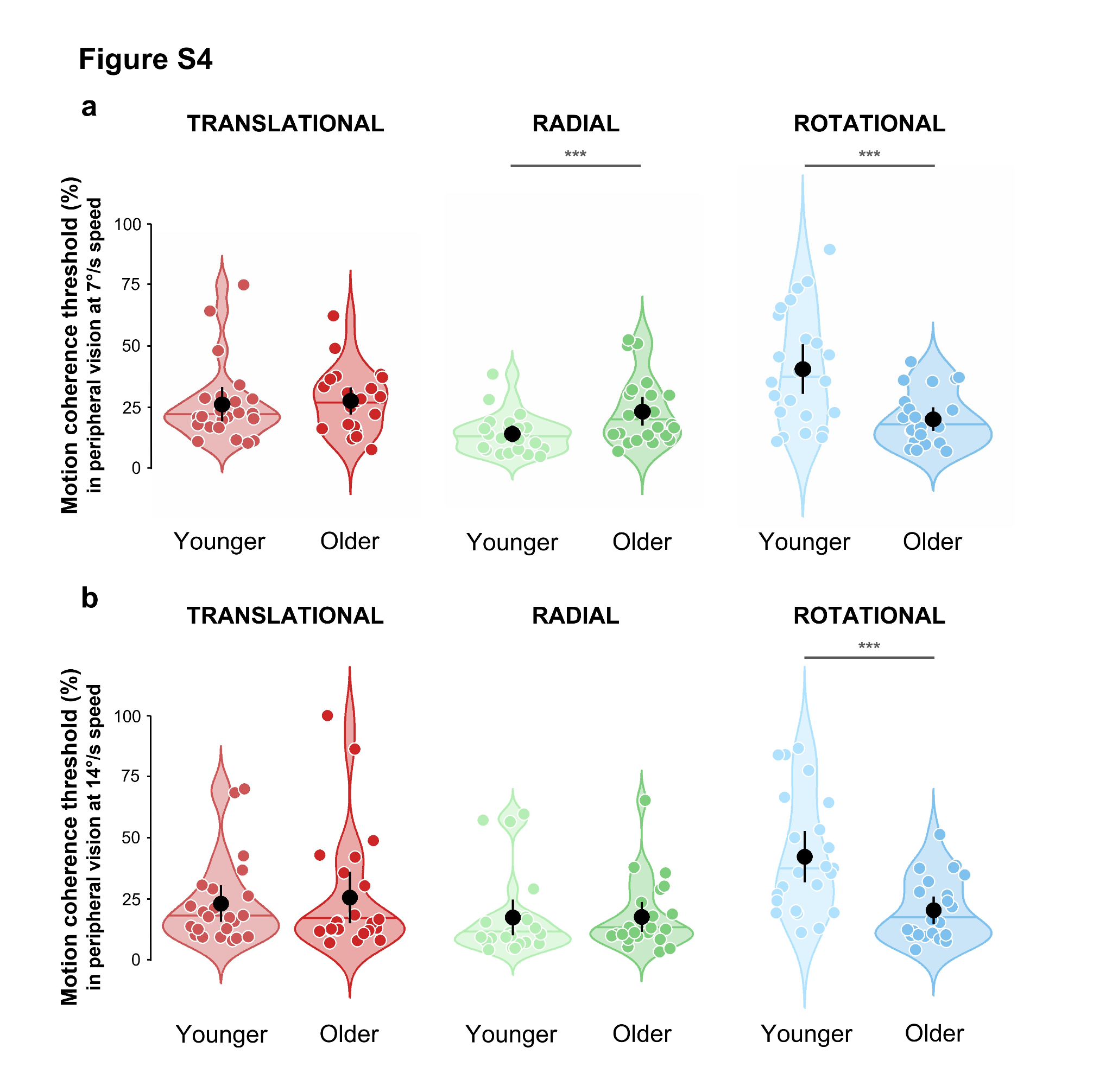
***

***Figure S4****. Distributions of the motion coherence thresholds estimated for translational (red), radial (green), and rotational (blue) optic flow patterns when central vision was masked and with speeds of either 7°/s (****a****) or 14°/s (****b****).See figure S1 for more details.*

**Supplementary Text S5: Reaction times**

Figure S5 shows the distributions of the data collected at a speed of 14°/s, with full-field stimuli (Fig. S5-A) and when central vision was masked (Fig. S5-B). Reaction times distributions in the two subgroups were similar with a speed of 7°/s.

**
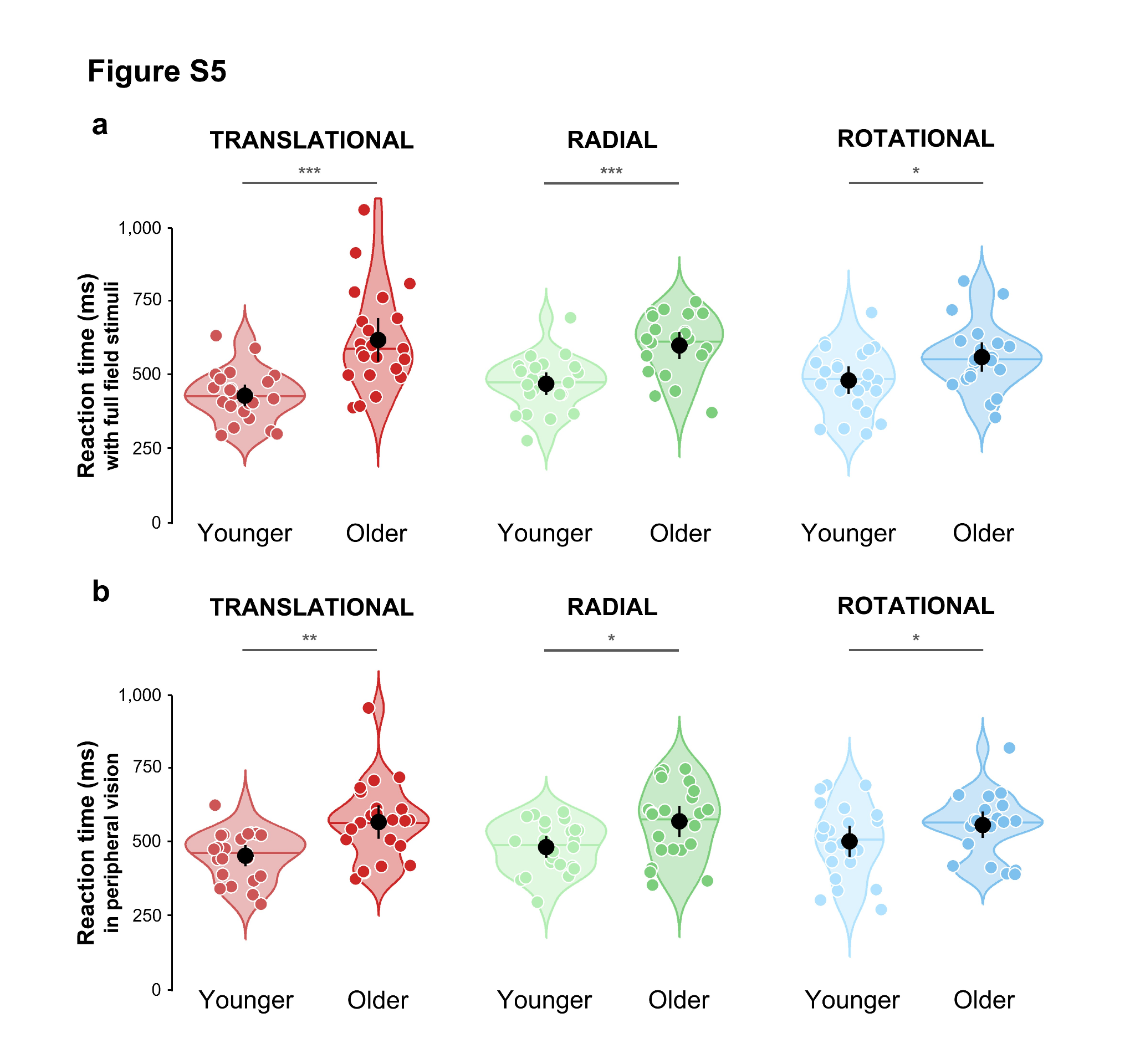
**

***Figure S5.*** *Reaction times estimated for translational (red), radial (green), and rotational (blue) optic flow patterns moving at 14°/s using full-field (****a****) or peripheral (****b****) stimuli.Stars indicate significantly different distributions ((***, p < .001; **, p < .01; * p < .05). See figure S1 for more details.*

**Supplementary Figure S6: Bias analysis**


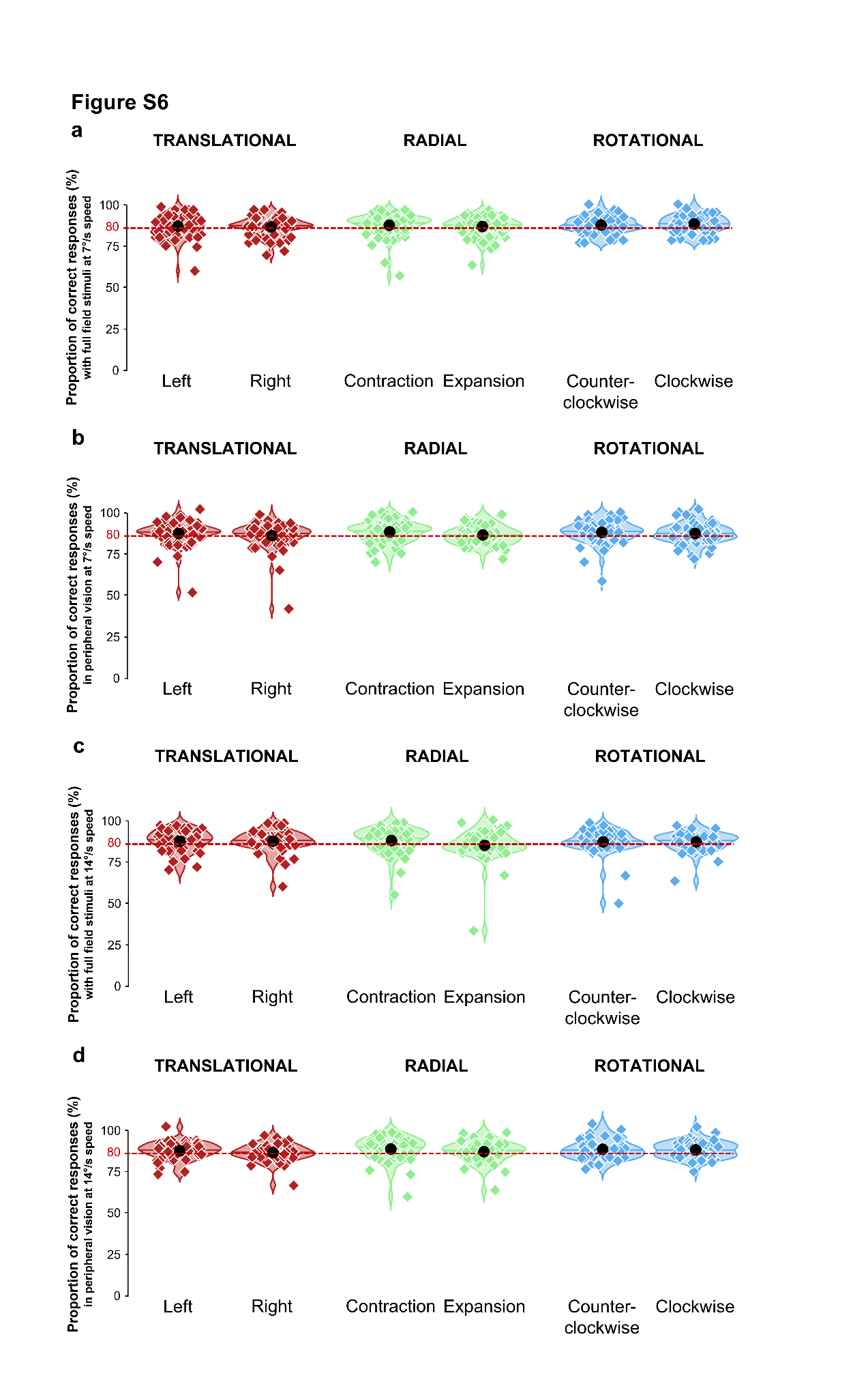


***Figure S6.*** *Proportion of correct responses for leftward and rightward motions (translational pattern), contraction and expansion (radial pattern), and counterclockwise and clockwise motions (rotational pattern). Red line corresponds to a performance level of 80%. Black dots represent means of proportion of correct responses for each direction of each optic flow pattern. Diamonds provide the individual data points of the distributions. Note that they were slightly offset horizontally to improve their visibility. Paired t-tests between the different data in controls and patients did not lead to any significant effects. Data provided here were obtained from (****a****) full-field stimuli moving at 7°/s, (****b****) peripheral stimuli moving at 7°/s, (****c****) full-field stimuli moving at 14°/s and (****d****) peripheral stimuli moving at 14°/s.*
